# Unilateral nevoid telangiectasia and ipsilateral capillary malformation

**DOI:** 10.1016/j.jdcr.2026.07.072

**Published:** 2026-08-25

**Authors:** Michelle D. Colbert, Jasmine Monge, Megha M. Tollefson

**Affiliations:** a Department of Dermatology, Mayo Clinic, Rochester, Minnesota; b Mayo Clinic Alix School of Medicine, Rochester, Minnesota; c Department of Pediatric and Adolescent Medicine, Mayo Clinic, Rochester, Minnesota

**Keywords:** capillary malformation, mosaicism, unilateral nevoid telangiectasia

## Introduction

Unilateral nevoid telangiectasia (UNT) is a rare vascular condition of the capillaries with fewer than 200 cases described.^1^ It may be congenital or acquired. Clinically, it presents with small telangiectasias surrounded by an anemic halo arranged in a unilateral Blaschkoid distribution on the face, neck, or upper chest.^2^ Telangiectasias may or may not be blanchable. Histopathology demonstrates increased capillary density in the papillary dermis.^3^ The proposed hypothesis of acquired UNT is that development is influenced by hormone levels and is therefore associated with puberty, pregnancy, liver disease, or exogenous hormone usage and has a 2:1 female-to-male ratio.^1^^,^^4^^,^^5^ Bier anemic spots, Becker nevus, and pyogenic granulomas have been reported in conjunction with UNT, but few other cutaneous associations have been observed.^6^ We present a case of UNT arising ipsilateral to a capillary malformation on the neck in the setting of liver cirrhosis.

## Case report

**Fig 1:**
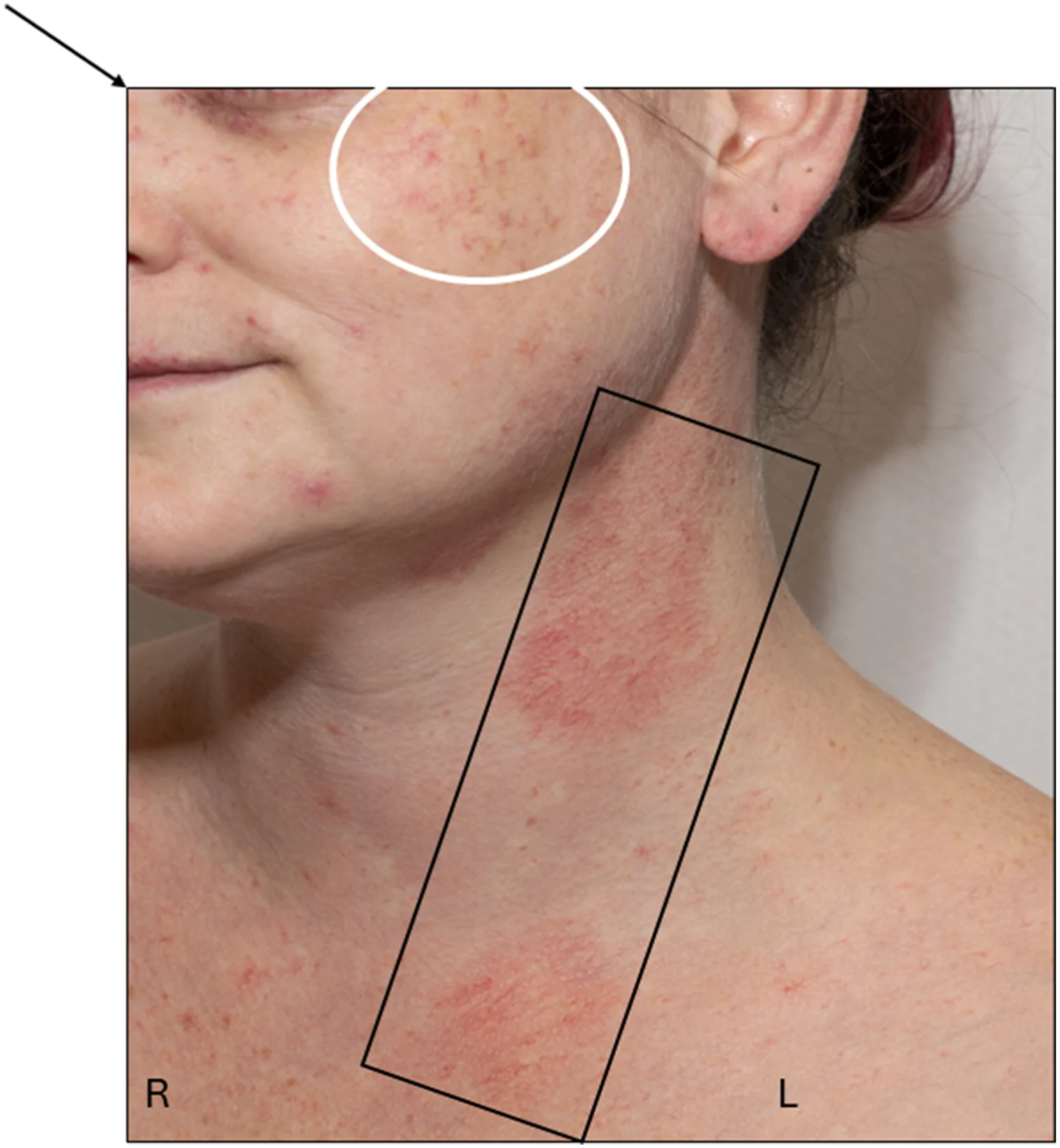
3/4 portrait demonstrating unilateral nevoid telangiectasia on the left cheek (*white circle*) and capillary malformation on the left lateral neck and upper chest (*black box*).

**Fig 2:**
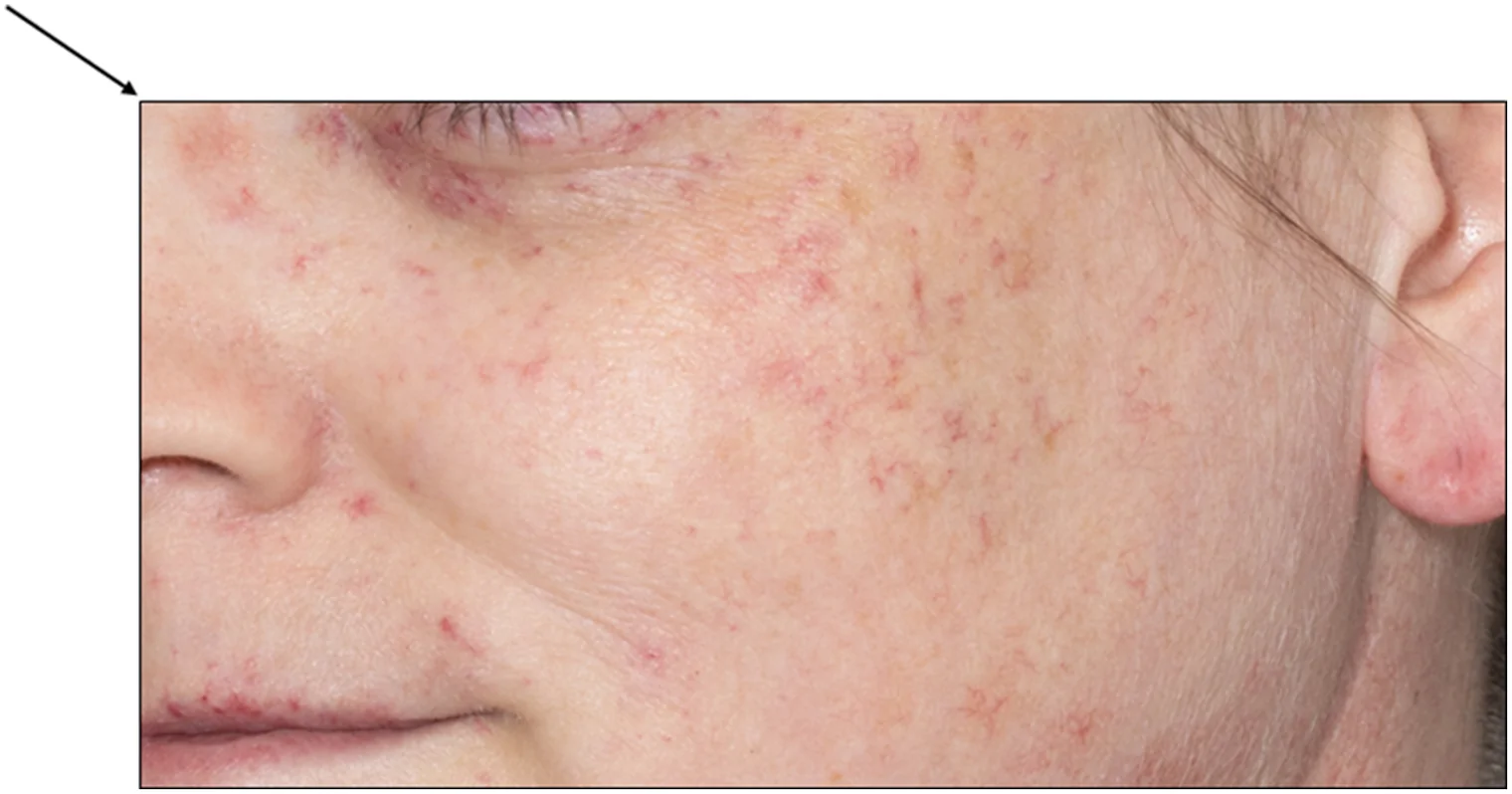
Confluence of telangiectasias involving the left lower eyelid, left earlobe, left cheek, vermilion border of the left upper lip and the mucosal lip.

The patient was a 35-year-old woman with a past medical history of alcohol-induced cirrhosis and hepatocellular carcinoma. She was referred for management of vascular lesions presumed to be secondary to cirrhosis. Physical examination revealed flat, ill-defined non-blanching red patches on the left side of the neck which were present since childhood and had not changed significantly over time, most consistent with a capillary malformation (CM) (Fig 1). There were discrete telangiectasias with anemic halos most confluent on the left hemiface, including the cheek, ear, and lip as well as the upper chest, which were new and increasing in number (Fig 2). These lesions were not blanchable and did not have a central arteriole with radiating vessels, thus were not consistent with spider angiomata.

The patient’s MELD-Na score was 6 at the time of cirrhosis diagnosis, which increased to 12 at the time of her Dermatology visit 4 years later. She had 2 prior pregnancies in 2005 and 2008 and did not develop similar telangiectatic lesions during or after these pregnancies. Given the distribution and clinical appearance of the new telangiectasias in conjunction with history of liver disease, a diagnosis of unilateral nevoid telangiectasia (UNT) in the setting of an ipsilateral CM was made. After discussion of the benign diagnosis, the patient decided not to treat.

## Discussion

We hypothesize that the capillary malformation represents localized vascular dysregulation and may have primed for the development of ipsilateral UNT. Involvement of areas such as the cheek, ear, and lip could be due to vascular dysregulation extending beyond the clinically apparent CM, which was limited to the neck. Vascular dysregulation was further exacerbated by hyperestrogenism in the setting of liver disease.

Capillary malformations are congenital vascular anomalies characterized by ectatic dermal capillaries which arise from post-zygotic somatic mosaic mutations, most commonly involving the *GNAQ* gene.^7^ These mutations are found in the majority of CMs and demonstrate low-level mosaicism limited to the affected tissues, supporting the concept of localized vascular dysregulation.

Given this established pathophysiology, somatic mosaicism has been proposed as a potential mechanism underlying the unilateral and Blaschkoid distribution of UNT,^8^ though a causative genetic mutation has not yet been identified. Blaschko lines represent a classical pattern of cutaneous mosaicism, reflecting populations of cells with distinct genetic profiles observable in congenital and acquired skin disorders.^9^ In certain conditions, mosaic populations of cells may exhibit altered responsiveness to systemic stimuli, leading to localized clinical manifestations. Kreft et al used laser-Doppler flowmetry to demonstrate the presence of morphologically altered capillaries in UNT, supporting the presence of structural microvascular change which may contribute to localized dysfunctional responses.^10^ Further, a mosaic pattern of estrogen-sensitive vascular endothelium has previously been proposed in UNT pathogenesis.^5^ Although the role of estrogen receptor overexpression in UNT remains debated, the effects of estrogen on vascular endothelium are well established especially in its role in regulation of angiogenesis and endothelial signaling.^11^

Hyper-estrogenic states are the most widely accepted mechanism for the development of UNT, and estradiol-induced stimulation of capillary endothelial cells is a well-established phenomenon.^8^ In liver disease, impaired hepatic metabolism of estrogen results in increased circulating estrogen levels, in turn promoting vascular dilation and proliferation. While spider angiomas are the most common cutaneous vascular manifestation of cirrhosis-induced hyperestrogenism, our patient’s lesions lacked the pathognomonic central feeding arteriole with radiating, blanchable vessels. Instead, her presentation of discrete, non-blanching telangiectasias confined to a unilateral Blaschkoid distribution was more consistent with a diagnosis of UNT.

Pregnancy-associated UNT is reported to have a waxing and waning course. Initial onset of UNT during a patient’s third pregnancy and increased number/wider distribution of telangiectasias in subsequent pregnancies have been observed.^8^^,^^12^^,^^13^ Development of UNT did not correspond with pregnancy status in our patient, but the UNT did progress as cirrhosis severity worsened as reflected by the increasing MELD-Na score. This temporal relationship may suggest hyperestrogenism in the setting of chronic hepatic dysfunction, rather than transient hormonal fluctuations in estrogen during pregnancy, as the main driver of UNT for this case.

In summary, we report an interesting case of unilateral nevoid telangiectasia arising near an ipsilateral capillary malformation. Cirrhosis is commonly associated with UNT, and progression of UNT in the setting of worsening cirrhosis was appreciated in our patient. Although the ipsilateral capillary malformation is an interesting finding, there is a lack of robust evidence to support a true association between capillary malformations and the development of UNT. This case provides a thoughtful discussion and review of localized vascular mosaicism in UNT pathogenesis.

## Conflicts of interest

None disclosed.
